# CPDDA: A Python Package for Discrete Dipole Approximation Accelerated by CuPy

**DOI:** 10.3390/nano15070500

**Published:** 2025-03-26

**Authors:** Dibo Xu, Paerhatijiang Tuersun, Shuyuan Li, Meng Wang, Lan Jiang

**Affiliations:** Xinjiang Key Laboratory for Luminescence Minerals and Optical Functional Materials, School of Physics and Electronic Engineering, Xinjiang Normal University, Urumqi 830054, China; 107622022210465@stu.xjnu.edu.cn (D.X.); wangm091999@stu.xjnu.edu.cn (M.W.); jianglan@mail.ustc.edu.cn (L.J.)

**Keywords:** discrete dipole approximation, light scattering, light absorption, graphics processing units, Python package

## Abstract

Discrete Dipole Approximation (DDA) is a rapidly developing numerical method in recent years. DDA has found wide application in many research fields including plasmonics and atmospheric optics. Currently, few DDA packages based on Python have been reported. In this work, a Python package called CPDDA is developed. It can be used to simulate the light-scattering and -absorption properties of arbitrarily shaped particles. CPDDA uses object-oriented programming, offers high flexibility and extensibility, and provides a comprehensive database of refractive indices. The package uses the biconjugate gradient method and fast Fourier transform for program acceleration and memory optimization, and it uses parallel computation with graphics processing units to enhance program performance. The accuracy and performance of CPDDA were demonstrated by comparison with Mie theory, the MATLAB package MPDDA, and the Python package pyGDM2. Finally, CPDDA was used to simulate the variations in light-absorption and -scattering properties of ZnO@Au core-shell nanorods based on the particle size. CPDDA is useful for calculating light-scattering and -absorption properties of small particles and selecting materials with excellent optical properties.

## 1. Introduction

The interaction of light with matter is a hot topic in physics. In recent years, the interaction of light with particles has emerged in applications in various fields such as biomedicine [1], atmospheric science [2], and sensing [3]. Although Mie theory can accurately and efficiently analyze such interactions, its applicability is limited to simple geometries like spheres. There are various numerical methods for calculating the light-scattering and -absorption properties of particles with arbitrary shapes. These include Finite Difference Time Domain (FDTD) [4], Finite Element Method (FEM) [5], Boundary Element Method (BEM) [6], the null-field method [7,8], and Discrete Dipole Approximation (DDA) [9]. Compared to FDTD and FEM, DDA is faster to compute. For the detailed characteristics of these numerical methods, see the published paper written by Amirjani and Sadrnezhaad [10].

DDA was originally proposed by Purcell and Pennypacker to calculate light scattering from grains in the interstellar medium [11]. Over the years, DDA has been applied to calculate the optical properties of arbitrarily shaped particles. In DDA, the particle is discretized into an array of *N* dipole points, and these polarizable points respond to the local electric field, thereby acquiring a dipole moment. For a finite array of point dipoles, the scattering problem can be solved exactly. Although DDA is simple to implement and feature-rich, it requires solving a 3*N* × 3*N* linear system of equations. For particles larger than the wavelength of the incident light, *N* becomes extremely large (close to a million), and the matrix becomes dense. Clearly, directly solving such a linear system is impractical. This problem can be solved using iterative methods and fast Fourier transform (FFT). In addition to reducing the computational complexity from *n*^2^ to *n*log(*n*), FFT also makes the interaction matrices sparse [12], which better satisfies the conditions for using iterative methods like Lanczos-Type Product Methods (LTPMs) [13].

Currently, several DDA open-source programs are available on the web. Firstly, DDSCAT [14], developed by Draine and Flatau in Fortran, has undergone significant growth and iteration over time. ADDA [15], written in C by Yurkin and Hoekstra, is a computational package designed for DDA calculations in cases involving high refractive indices. It supports OpenCL hybrid programming, enabling the use of graphics processing units (GPUs) to accelerate the computations. Donald et al. developed OpenDDA [12] using C, and it provides two parallel computation modes: OpenMP and MPI. Loke et al. wrote DDA-SI [16] using MATLAB, which can calculate the case involving surface interactions. However, FFT and iterative methods are not used in it to speed up and reduce program memory. Shabaninezhad et al. developed a MATLAB package called MPDDA [17], which enables parallel acceleration through GPU. Chaumet et al. implemented IF-DDA [18] using Fortran with a graphical user interface (GUI), allowing the computation of interactions between inhomogeneous, anisotropic objects within multilayer structures. Additionally, Chaumet compared the performance of various iterative methods in DDA [19] and tested two methods called Induced Dimension Reduction and Generalized Product-Bi-conjugate Gradient Stabilized, both of which had never been applied to DDA before.

It is a clear trend in computational sciences over the last two decades that researchers tend to move from low-level to high-level languages like MATLAB and Python. These languages allow for fast algorithm implementation and are easy to read, attracting more researchers and simplifying code iteration. High-performance computing libraries in C/C++, such as BLAS and LAPACK, can be directly called from Python. With just a few lines of code, Python can achieve computation speed comparable to multi-line compiled C/C++ code. For example, NumPy [20] is a library widely used for linear algebra and array operations. Additionally, GPUs can bypass the limitations of Python’s Global Interpreter Lock (GIL) to effectively accelerate the CPU-bound part of code. If DDA code can be easily implemented in Python without significant loss of computational efficiency, it could open up a new perspective for developing DDA packages in Python. It is worth noting that another volume integral method similar to DDA, known as the Green Dyadic Method (GMD) [21,22], has also been implemented in Python. However, unlike DDA, the GMD method relies on the environment for its self-term, while the self-term of DDA depends on the discrete object itself. Furthermore, DDA incurs less overhead when computing anisotropic media. It is worth mentioning that the GDM python package pyGDM2 [22] and the null-field method python package SMUTHI [23] both have GPU acceleration.

In this paper, we have developed a Python package called CPDDA, which is available for free download on GitLab (https://gitlab.com/dmcxu1/cpdda (accessed on 21 March 2025)). CPDDA is object-oriented and provides two modes of operation: CPU and GPU. The GPU mode offers significantly higher computational efficiency compared to the CPU mode. In addition, the package includes a comprehensive database of refractive indices (3107 experimental data for 398 materials), which can offer powerful assistance for researching and optimizing the optical properties of various particles. Finally, the light-absorption and -scattering properties of ZnO@Au core-shell nanorods were investigated using CPDDA as an example.

## 2. Fundamentals of DDA

DDA is a numerical method based on volume-integral discretization derived directly from Maxwell’s equations [14]. It calculates optical properties like absorption, scattering, and extinction cross sections for particles of any shape and composition. The method approximates a continuous target by a finite array of polarizable points. The target is discretized into *N* points, each represented as a dipole and periodically arranged on a cubic lattice with a side length of *d*. The points acquire dipole moments in response to the local electric field. Therefore, the only approximation in the DDA is replacing the continuum target with an array of *N*-point dipoles.

Assuming the center coordinates of each dipole are {**r*_p_***, *p* = 1, 2, …, *N*}, and the polarizability is {α*_p_*, *p* = 1, 2, …, *N*}, each dipole generates a dipole moment under the influence of the local electric field:

(1) $$P_{p}=\alpha_{p}E_{p},$$

where **E*_p_*** is the electric field at **r***_p_* due to the incident wave **E**_inc,_*_p_* plus the contribution of each of the other *N* − 1 dipoles:

(2) $$E_{p}=E_{\mathrm{inc},p}-\sum_{q\neq p}A_{pq}P_{q},$$

where **A***_pq_* is the 3 × 3 interaction matrix containing the Green tensor, which can be expressed as:

(3) $$A_{pq}=\frac{\exp(ikr_{pq})}{r_{pq}}\times \left[\left.k^{2}\left({\hat{r}}_{pq}{\hat{r}}_{pq}-I_{3}\right)+\frac{ikr_{pq}-1}{r_{pq}^{2}}\left(3{\hat{r}}_{pq}{\hat{r}}_{pq}-I_{3}\right)\right]\right.,\;\;p\neq q,$$

where k=ωc is the wave number, $r_{pq}=\;|r_{p}-r_{q}|$ is the distance from dipole *q* to *p*, ${\hat{r}}_{pq}=(r_{p}-r_{q})/r_{pq}$ is a unit vector in the direction from dipole *q* to *p*, and **I**_3_ represents the 3 × 3 identity matrix. The diagonal elements of the interaction matrix are defined as $A_{pp}={\alpha_{p}}^{-1}I_{3}$, which leads to the simplified form of Equation (2) as:

(4) $$\sum_{q=1}^{N}A_{pq}P_{pq}=E_{\mathrm{inc},p}.$$

Equation (4) is the final linear equation system to be solved and the primary objective of DDA. Once Equation (4) has been solved for the unknown polarization **P***_p_*, the extinction, absorption, and scattering cross sections can be evaluated:

(5) Cext=4πkE02∑p=1NIm(Einc,p∗⋅Pp),

(6) Cabs=4πkE02∑p=1NIm[Pp⋅(αp−1)∗Pp∗]−23k3|Pp|2,

(7) $$C_{\mathrm{sca}}=C_{\mathrm{ext}}-C_{\mathrm{abs}}.$$

After discretizing the target object into dipoles, each dipole can be considered to represent a specific polarizability of the target’s material and can interact with other *N* − 1 dipoles. The dielectric properties of a material are directly linked to the polarizability. When dipoles are arranged on an infinite cubic lattice, this relationship is described by the simple and precise Clausius–Mossotti relation:

(8) $${\alpha_{p}}^{CM}=\frac{3d^{3}}{4\pi }\frac{\epsilon_{p}-1}{\epsilon_{p}+2},$$

where *ϵ_p_* is the dielectric function of the target’s material at location **r***_p_*. However, the derivation of this correction was based on the assumption that the electric field remains uniform across cubic regions with a volume of *d*^3^. Thus, we consider the Lattice Dispersion Relation (LDR) polarizability. Since the dipoles are arranged on a periodic cubic lattice and coupling occurs between the electromagnetic waves and the lattice, consider that for the LDR polarizability *α*, the infinite lattice of polarizable points has the same dispersion relation as the continuous refractive index *m*. In the limit *kd* << 1, the polarizability *α* is expressed as a power series expansion in terms of *kd* and *m*^2^ = ϵ:

(9) $$\alpha^{\mathrm{LDR}}\approx \frac{\alpha^{\mathrm{CM}}}{1+(\alpha^{\mathrm{CM}}/d^{3})[(b_{1}+b_{2}m^{2}+b_{3}m^{2}S){\left(kd\right)}^{2}-(2/3)i{\left(kd\right)}^{3}]},$$

where:

(10) $$b_{1}=-1.891531,\;\;\;\;b_{2}=0.1648469,\;b_{3}=-1.7700004,\;\;\;\;S=\sum_{j=1}^{3}{\left({\hat{a}}_{p}{\hat{e}}_{p}\right)}^{2}.$$

In order to ensure the accuracy of the DDA calculation results, the rule of the following equation is followed:

(11) kd|m|<<1.

For irregular particles, the criteria may be appropriately adjusted to account for their specific characteristics [24]. The subsequent section describes the implementation of DDA in Python, including acceleration techniques, and provides a quantitative assessment of the code’s accuracy, reliability, and performance.

## 3. Implementation and Validation of CPDDA

### 3.1. Implementation of CPDDA

**Figure 1:**
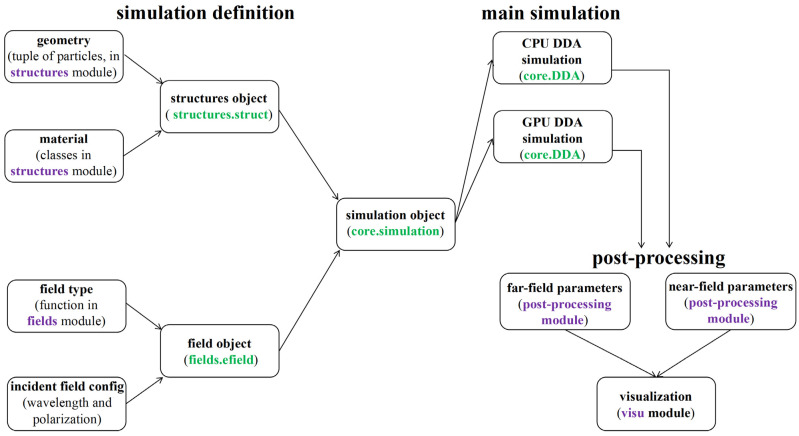
Structure of the CPDDA package and workflow of the typical simulation: (1) Setup of the geometry, material, and incident electric field. They are bundled in an instance of the simulation object. (2) Main simulation. (3) Post-processing. (4) Visualization of the results.

Our primary algorithms are based on MPDDA, and we have extended this framework to develop CPDDA using Python. CPDDA uses an object-oriented form to program the DDA. The structure of the CPDDA package and the main steps to set up and run a simulation are schematically depicted in Figure 1. The core of CPDDA is the simulation object, which contains information about the structure and the incident field used in the simulation. In Appendix A, a simple Python script for CPDDA to create a simulation object is supplied. The geometry of the particle and the refractive indices of the particle and the environment are stored in an instance of structures.struct. The geometry module is used to establish the grid coordinates of the particle, and the materials object contains the dipole material properties, including the refractive index of the particles and the corresponding dielectric constant. It is worth noting that CPDDA contains a rich database of refractive indices of materials, which provides 398 kinds of materials (including 3107 kinds of experimental data). Table 1 presents a summary of the database. The diverse range of refractive indices in this package’s database enables researchers to explore novel optical properties of materials and conduct comparative studies within material classes to identify high-performance candidates for specific applications. After setting up the first two objects, the polarizability of each dipole is determined by placeholders.

The second key ingredient of CPDDA simulation is the incident field. The part about the incident field is set in the field.efield object, which contains the propagation direction and polarization of the incident field. The current CPDDA implementation contains two incident fields: plane wave and Gaussian beam.

The above two parts together set up and define the simulation object, thus setting **E**_inc,*p*_ and the diagonal elements in **A***_pq_* in Equation (4). The main simulation of CPDDA is divided into constructing matrix–vector multiplications and solving them. For Equation (4), it can be written as follows:

(12) $$\left[\begin{array}{ccc} A_{xx} & A_{xy} & A_{xz}\\ A_{yx} & A_{yy} & A_{yz}\\ A_{zx} & A_{zy} & A_{zz} \end{array}\right]\left[\begin{array}{c} P_{x}\\ P_{y}\\ P_{z} \end{array}\right]=\left[\begin{array}{c} E_{\mathrm{inc},x}\\ E_{\mathrm{inc},y}\\ E_{\mathrm{inc},z} \end{array}\right],$$

where $A_{\mu \nu }$ (μ and ν is *x*, *y*, or *z*) is an *N*×*N* matrix that represents the interaction matrix, and $P_{\mu }$ and $E_{\mathrm{inc},\mu }$ (μ and ν is *x*, *y*, or *z*) are denoted as the three polarization vectors of *N*×1 and the three components of the incident electric field, respectively. Since the nine blocks of the interaction matrix are symmetric regarding changing the indices, only six independent blocks of *A* (*A_xx_*, *A_xy_*, *A_xz_*, *A_yy_*, *A_yx_*, *A_zx_*) are required.

After constructing the matrix, it is time to solve for this matrix–vector multiplication. Due to the size of the matrix, it takes a significant amount of computing resources to generate the interaction matrix’s inverse, and solving Equation (12) in this case is not possible. Therefore, the solution is to use an iterative method to solve the linear system. The principle of an iterative method for solving the linear system is to create a sequence **E*_s_*** such that:

(13) $$r_{q}=\frac{∥\bar{A}E_{q}-E_{\mathrm{inc},p}∥}{∥E_{\mathrm{inc},p}∥},$$

with the residue *r_q_* tending towards zero when *q* increases, in which the *q*-th approximation is derived from the previous ones. The iterative process is stopped when *r_q_* < *η*, where the value of *η* is set by the user and depends on the desired precision of the field [13,19]. Bi-conjugate gradient (Bi-CG) has been employed in several DDA implementations [12,17], and CPDDA also adopts this iterative method.

The iterative method involves several matrix–vector multiplications, which are further accelerated by 3D FFT. The interaction matrix is constructed as a Toeplitz matrix, where all diagonal elements are equal, and the off-diagonal elements along each parallel diagonal are equal. Thus, the Toeplitz matrix can be entirely defined by its first row and first column as [25]:

(14) $$T_{n}=[t_{q,p};q,p=0,\;1,\;\cdots ,\;n-1].$$

By setting *t_q,p_* = *t_q-p_* and appending the first column (excluding the initial term) in reverse order to the first row, then completing the diagonal, the Toeplitz matrix is transformed into the circulant matrix $C^{n^{\prime }}$, where $n^{\prime }=2n-1$. Arbitrary circulant matrices can be diagonalized using Fourier matrices:

(15) $$F^{n^{\prime }}=\frac{1}{\sqrt{n^{\prime }}}\omega^{pq},\;p,\;q=0,\;\cdots ,\;n^{\prime }-1,\;\omega =e^{-\frac{2\pi i}{n^{\prime }}},$$

(16) $$F^{n^{\prime }}C^{n^{\prime }}F^{H}=diag(\sqrt{n^{\prime }}F^{n^{\prime }}C^{n^{\prime }})=diag(\lambda_{c}).$$

Equation (15) is the expression for the Fourier matrix using the fact that the Fourier matrix is unitary, F−1[n′]=FH[n′]. Where the H superscript is the transposed conjugate, the multiplication of an arbitrary vector with a circulant matrix can be performed as an element-wise multiplication in the Fourier domain via the Convolution Theorem. For a comprehensive discussion of specific algorithms for iterative methods and FFT, readers may refer to the studies by Shabaninezhad et al. and Donald et al. [12,17]. All of the above operations for matrix–vector multiplication can be found in the core.DDA module. core.DDA is divided into CPU and GPU compute modes. The next section details how CPU-bound prat blocks are accelerated in GPU mode.

After calculating the polarization vector **P*_p_***, CPDDA can compute the near-field and far-field parameters and visualize them through the visu module. Specifically, the three main cross-sections, efficiency and volume coefficients, and the electric field distribution around the particles can be calculated; the corresponding code is available in the post-processing module.

### 3.2. GPU Mode of CPDDA

For CPU-bound tasks like DDA, parallel computing is an efficient computational method. Due to the GIL, explicit multi-threading cannot be implemented in Python to achieve parallelism. Using multi-threading through tools like Cython limits programming to basic atomic operations, which prevents using the high-performance capabilities of libraries like NumPy and increases programming complexity [26,27]. Therefore, GPU mode is added to core.DDA for parallel computation.

**Figure 2:**
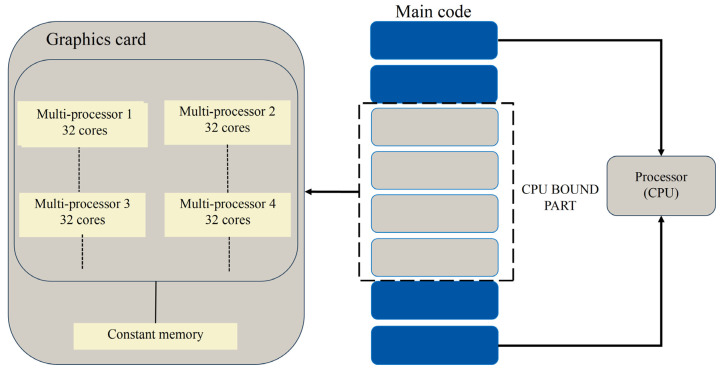
Schematic diagram of CPDDA data transfer from CPU to GPU.

In general, GPUs have thousands of cores and are capable of running several thousands of threads simultaneously. Although modern CPUs can have an even larger number of cores, they are still very small compared to GPUs. Since the GPU is a foreign device compared to the host CPU, any data that need to be processed on a GPU must be offloaded to it first. The data transfer between CPU and GPU is performed via a PCIe cable. PCIe data communication can be bottlenecked by large data transfers, and in extreme cases, data transfer costs may exceed computation times. Therefore, considering the hardware communication overhead, only the CPU-bound part of the code is offloaded to the GPU, as shown in Figure 2. CPU-bound iterative methods and matrix–vector multiplications were offloaded to the GPU, with only two communications between the CPU and GPU during the entire computation. In the Python implementation, we used CuPy and NumPy for high-performance computing and transfered data between the CPU and GPU using the cp.array and cp.asnumpy. Additionally, the memory pooling technique in CuPy manually replaces some of Python’s garbage collection techniques for more efficient memory management. It is worth mentioning that the CuPy library offers a highly compatible interface with NumPy, enabling GPU-accelerated data processing and scientific computing, particularly for large-scale data. GPU acceleration is available when the user selects GPU mode in the core.DDA module.

### 3.3. Numerical Validation of CPDDA

**Figure 3:**
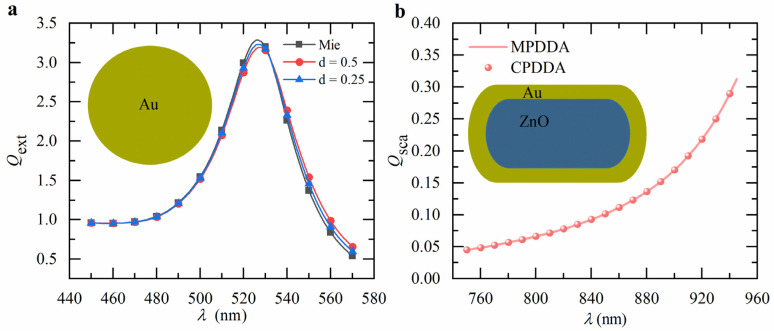
(**a**) Calculated extinction spectra of Au nanosphere using Mie theory and CPDDA (lattice spacing is 0.5 nm and 0.25 nm, respectively). (**b**) Calculated scattering spectra of ZnO@Au nanorod using MPDDA and CPDDA. In the simulation, the diameter of Au nanosphere is 30 nm, and core length, shell thickness, and aspect ratio of core-shell nanorod are 60 nm, 5 nm, and 12, respectively. The refractive indices of the environment are (**a**) 1.33 and (**b**) 1.44, respectively.

To verify the accuracy of CPDDA, the calculations were performed using a LAPTOP-3EE0017T with Windows 10 operating system. The laptop was equipped with an Intel(R) i5-7300HQ processor with 2.50 GHz and 8192 MB of total RAM as well as a NVIDIA GeForce RTX 1050ti graphics card with 4096 MB of RAM, and Python 3.10 with 13.1.0 cupy-cuda12x was installed on the laptop. We calculated the extinction efficiency of the Au nanosphere with a diameter of 30 nm in an aqueous solution for two lattice spacings, *d* = 0.5 nm and 0.25 nm, and compared the results with the Mie theory using PyMieLab_V1.0 [28], as shown in Figure 3a. The CPDDA results closely matched Mie theory with a relative error of less than 2% for *d* = 0.25 nm, and the spectra converged to the exact solution as the lattice spacing was reduced. Additionally, scattering efficiency of ZnO@Au nanorods, characterized by a core length of 60 nm, a shell thickness of 5 nm, and an aspect ratio of 12, were calculated and compared with the results from MPDDA [17], as shown in Figure 3b. The results are in excellent agreement, demonstrating the accuracy and reliability of CPDDA.

**Figure 4:**
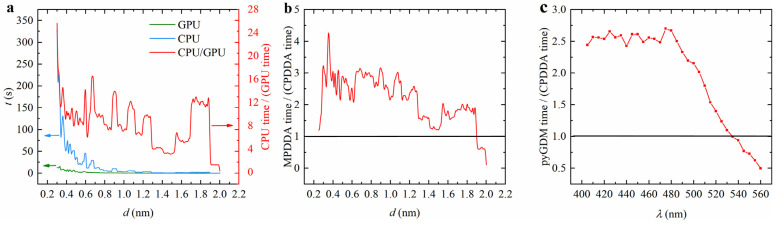
(**a**) Variation of CPU and GPU computation time and their ratio with the lattice spacing *d*. (**b**) Variation of the ratio of MPDDA to CPDDA computation time with the lattice spacing *d*. In the simulation, the particle is an Au nanocube with an isomeric diameter of 40 nm, the refractive index of the environment is 1.33, and the incident light is a plane wave with a wavelength of 450 nm. (**c**) Variation of the ratio of pyGDM2 to CPDDA computation time with the wavelengths *λ,* the diameter of Au nanosphere is 50 nm, the number of discrete units for both is around 1000.

To demonstrate the GPU acceleration of CPDDA, we compare the computation time for the CPU and GPU under the same configurations. Figure 4a shows the variations between the CPU and GPU computation times with lattice spacing *d* for an Au nanocube with an isomeric diameter of 40 nm in a medium with a refractive index 1.33 and incident light wavelength of 450 nm. As *d* decreases, the GPU acceleration becomes more pronounced. As the lattice pitch decreases, CPU computation time increases significantly, while GPU computation time remains relatively constant. At *d* = 0.3 nm, CPU time is approximately 26 times that of the GPU. To further evaluate performance, we compared the GPU mode of CPDDA with that of MPDDA on the same configured system, both using Bi-CG as the iterative solver. The computation time ratio between MPDDA and CPDDA demonstrates the acceleration advantage of CPDDA, as shown in Figure 4b. The results indicate that CPDDA is generally faster, but MPDDA outperforms CPDDA when d reaches 1.9 nm. CPDDA shows a computational speed advantage at smaller lattice spacings. To further evaluate the computational efficiency, we compared the performance of pyGDM2 and CPDDA in GPU mode. As Figure 4c shows, we simulated Au nanospheres (50 nm diameter in a vacuum) using both pyGDM2 and CPDDA under GPU acceleration based on the example provided in the pyGDM2 documentation. Due to the high memory demand of pyGDM2 compared to DDA for the same number of discrete cells, the analysis was limited to approximately 1000 discrete units. The results demonstrate that while CPDDA exhibits superior performance at shorter wavelengths, pyGDM2 outperforms at longer wavelengths.

## 4. Optical Properties of ZnO@Au Nanorods

ZnO@Au core-shell nanoparticles, consisting of ZnO core and Au shell, show significant potential in applications such as surface-enhanced Raman scattering, photocatalysis, and biosensing [29,30,31,32]. ZnO, a semiconductor with a bandgap of 3.37 eV at room temperature, exhibits good electrical conductivity, making it valuable in electrochemical devices [33]. Moreover, these nanoparticles are low in toxicity, biodegradable, and easy to synthesize [34]. When coated with Au, ZnO@Au nanoparticles exhibit enhanced biocompatibility. ZnO@Au core-shell nanorods have been synthesized and widely used in gas sensing [35], and other fields [36]. In this section, ZnO@Au core-shell nanorods are used to quantitatively analyze the variation in their light-absorption and -scattering properties with particle size using CPDDA. The refractive index data of Au and ZnO were obtained from ref. [37] and ref. [38], respectively, and the refractive index of the surrounding medium was set to 1.44 (representing subcutaneous fat) [39]. The light-absorption and -scattering characteristics were quantified by the volume-absorption coefficient *A*_abs_ and volume-scattering coefficient *A*_sca_, defined as follows:

(17) $$A_{\mathrm{abs}}=C_{\mathrm{abs}}/V_{p},$$

(18) $$A_{\mathrm{sca}}=C_{\mathrm{sca}}/V_{p},$$

where *V*_p_ represents the overall volume of the particle (including the shell).

**Figure 5:**
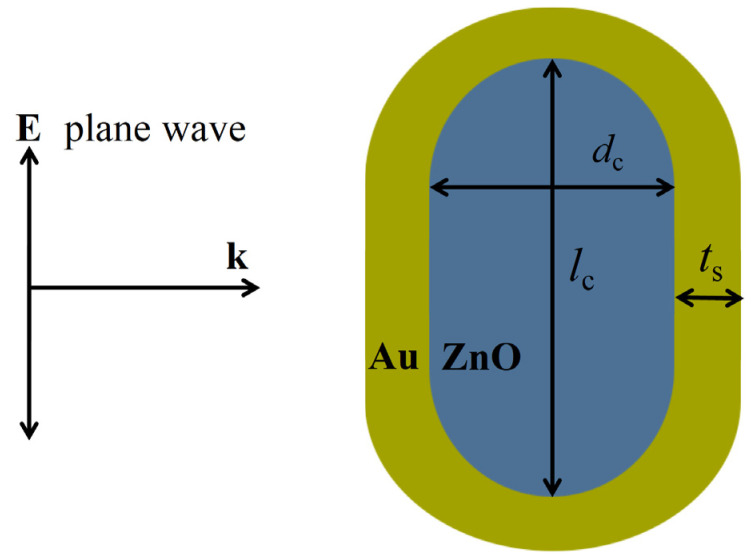
Schematic of the interaction of ZnO@Au core-shell nanorod with light.

The schematic diagram of interaction of light with the ZnO@Au core-shell nanorod is shown in Figure 5. The ZnO@Au core-shell nanorod is represented by three geometrical parameters, where *l*_c_ denotes the core length, *d*_c_ denotes the core diameter, and *t*_s_ denotes the shell thickness. The ratio of core length to diameter is defined as the aspect ratio *AR*, and the particle geometry can also be expressed in terms of *AR*, *t*_s_, and *l*_c_. The incident light propagates along the short axis of the nanorod, and the electric field is polarized along the long axis of the nanorod.

### 4.1. Effect of Aspect Ratio

**Figure 6:**
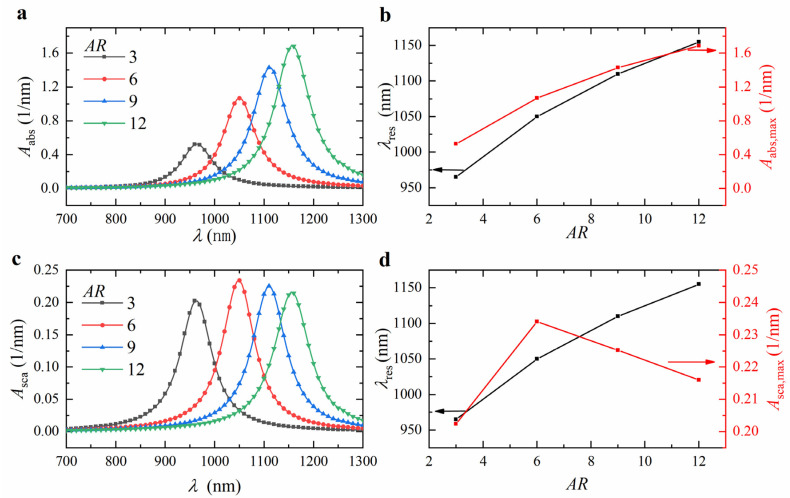
Calculated (**a**) absorption and (**c**) scattering spectra of ZnO@Au core-shell nanorods with different aspect ratios *AR*. Variation of (**b**) absorption and (**d**) scattering resonance wavelength and peak intensity with *AR*. The core length *l*_c_ and shell thickness *t*_s_ were 80 nm and 5 nm, respectively.

To quantitatively analyze the effect of the core aspect ratio *AR* on the absorption and scattering spectra, we calculated the spectra for ZnO@Au core-shell nanorods with *AR* values of 3, 6, 9, and 12, fixing the core length *l*_c_ at 80 nm and the shell thickness *t*_s_ at 5 nm. The results are shown in Figure 6a,c. As the *AR* increased from 3 to 12, the resonance wavelength redshifted from 965 nm to 1055 nm (see Figure 6b,d), the volume-absorption coefficient increased from 0.5256 nm^−1^ to 1.6854 nm^−1^ (see Figure 6b), and the volume-scattering coefficient first increased from 0.2024 nm^−1^ to 0.2340 nm^−1^, then decreased to 0.2160 nm^−1^ (see Figure 6d). The imaginary part of the dielectric function is expected to exhibit an increasing trend with wavelength, as the collision frequency of electrons in the metal becomes comparable to the optical frequency in the lower energy (longer wavelength) regime. At these wavelengths, the absorption contribution is anticipated to rise due to the enhancement of the metal’s intrinsic dielectric function. This effect is particularly pronounced for Au, where the increase in the imaginary dielectric function is more significant. This could be the reason for the decrease in the maximum value of the bulk-scattering coefficient at longer wavelengths for high-aspect-ratio nanorods [40]. The optical properties and electronic structure of the nanoparticles changed with *AR*. As the *AR* increased, the charge accumulation on the nanoparticle surface decreased, weakening the electron-restoring force and lowering the Local Surface Plasmon Resonance (LSPR) frequency, which resulted in a redshift of the resonance wavelength [41].

### 4.2. Effect of Shell Thickness

**Figure 7:**
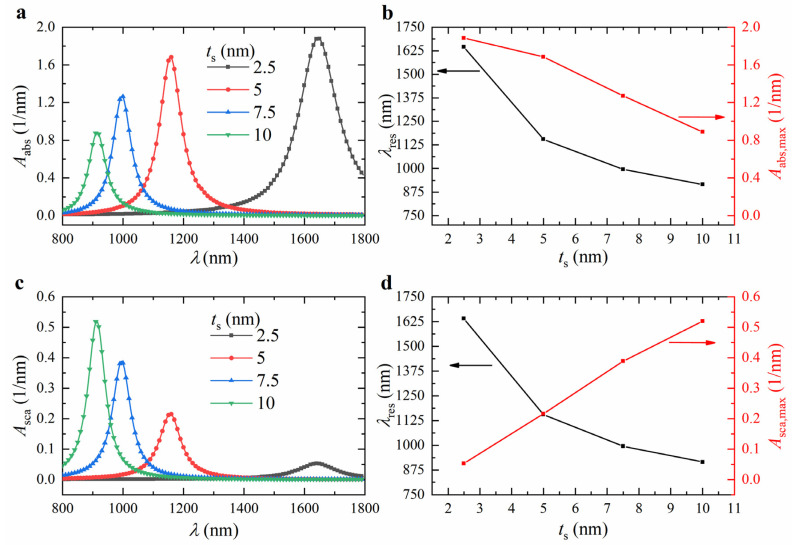
Calculated (**a**) absorption and (**c**) scattering spectra of ZnO@Au core-shell nanorods with different shell thicknesses *t*_s_. Variation of (**b**) absorption and (**d**) scattering resonance wavelength and peak intensity with *t*_s_. The core length *l*_c_ and aspect ratio *AR* are 80 nm and 12, respectively.

To further analyze the effect of the shell thickness *t*_s_ on the absorption and scattering spectra, we calculated the spectra for ZnO@Au core-shell nanorods with *t*_s_ values of 2.5, 5, 7.5, and 10 nm, fixing the core length *l*_c_ at 80 nm and the core aspect ratio *AR* at 12. The results are shown in Figure 7a,c. As the *t*_s_ increased from 2.5 to 10 nm, The resonance wavelength of the ZnO@Au core-shell nanorods underwent a significant blueshift (see Figure 7b,d), the volume-absorption coefficient decreased from 1.8834 nm^−1^ to 0.8885 nm^−1^ (see Figure 7b), and the volume-scattering coefficient increased from 0.0521 nm^−1^ to 0.5204 nm^−1^ (see Figure 7d). As *t*_s_ increased, the accumulated surface charge on the nanoparticle enhanced the electron-restoring force, raising the LSPR frequency and inducing a blueshift in the resonance wavelength.

### 4.3. Effect of Core Length

**Figure 8:**
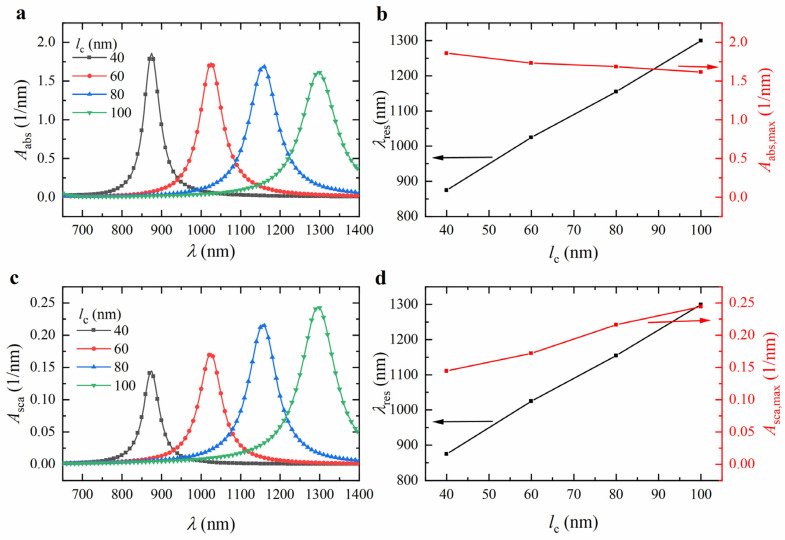
Calculated (**a**) absorption and (**c**) scattering spectra of ZnO@Au core-shell nanorods with different core lengths *l*_c_. Variation of (**b**) absorption and (**d**) scattering resonance wavelength and peak intensity with *l*_c_. The shell thickness *t*_s_ and aspect ratio *AR* were 5 nm and 12, respectively.

To further analyze the effect of the core length *l*_c_ on the absorption and scattering spectra, we calculated the spectra for ZnO@Au core-shell nanorods with *l*_c_ values of 40, 60, 80, and 100 nm, fixing the shell thickness *t*_s_ at 5 nm and the core aspect ratio *AR* at 12. The results are shown in Figure 8a,c. As the *l*_c_ increased from 40 to 100 nm, the resonance wavelength redshifted from 875 nm to 1300 nm (see Figure 8b,d), the volume-absorption coefficient decreased from 1.8589 nm^−1^ to 1.6166 nm^−1^ (see Figure 8b), and the volume-scattering coefficient increased from 0.1444 nm^−1^ to 0.2440 nm^−1^ (see Figure 8d). As *l*_c_ increased, the vibrational distance of free electrons within the Au shell increased, decreasing their vibrational frequency and lowering the LSPR frequency, which induced a redshift in the resonance wavelength.

## 5. Conclusions

This paper presents CPDDA, a Python package for simulating the light-scattering and -absorption properties of arbitrarily shaped particles. CPDDA uses Bi-CG and FFT algorithms to accelerate the CPU-bound part of DDA and reduce memory usage. To improve performance and overcome GIL limitations, CuPy was used to implement parallel computation on the GPU, significantly enhancing computation speed compared to the CPU. Having a rich database of refractive indices of materials (3107 refractive index experimental data of 398 materials) is one of the features of this package, which can provide powerful help for studying and optimizing electromagnetic properties of particles with multiple materials. Compared to Mie theory, MPDDA, and pyGDM2, the reliability and performance of CPDDA were validated. Finally, ZnO@Au core-shell nanorods were used to study how their light-absorption and -scattering properties varied with aspect ratio, shell thickness, and core length. The high-level Python API, along with various tools for rapid data analysis and visualization, renders standard optical simulations of particles relatively straightforward. In the future, more optical parameters will be added to CPDDA to expand the functionality of the package, and more high-performance algorithms will be integrated to support broader applications.

## Figures and Tables

**Table 1:** Description of material refractive index database.

Material Classification	Description
MAIN	simple inorganic materials
GLASS	glasses
ORGANIC	organic materials
OTHER	miscellaneous materials

## Data Availability

The original contributions presented in this study are included in the article; further inquiries can be directed to the corresponding authors.

## Appendix A. A CPDDA Code Example for Setting the Simulation Object

import numpy as np from CPDDA import materials from CPDDA import structures From CPDDA import fields from CPDDA import core from CPDDA import post_processing d = 2 r_eff = 20 wavelength = np.arange(450, 570, 5, dtype=np.float32) material1=materials.FromDatabase(shelf_name="main",book_name="Au", page_name="Johnson.yml",wl=wavelength, nb=1.33) material = [material1] geometry = structures.sphere(r_eff, d) occupied = structures.INDEX_in("spherical", geometry) struct = structures.struct(d, material, occupied, geometry) print(struct) field_generator = fields.plan_wave() K_kwargs = dict(K0=np.array([1, 0, 0])) E_kwargs = dict(E0=np.array([0, 0, 1])) efield = fields.efield(field_generator, wavelength, K_kwargs, E_kwargs) print(efield) sim = core.simulation(struct, efield)
